# Impact of Endodontic Treatment of Teeth With Apical Periodontitis on Levels of Inflammatory Biomarkers Associated With Cardiovascular Risk: A Systematic Review and Meta‐Analysis

**DOI:** 10.1155/tswj/5896031

**Published:** 2026-04-23

**Authors:** Carolina Viana Vasco Lyra, Jéssica Meirinhos Miranda, Renata de Albuquerque Cavalcanti Almeida, Marina da Cunha Isaltino, Ana Virginia Silva Vilela, Marleny Elizabeth Márquez de Martínez Gerbi, Diana Santana de Albuquerque, Natália Gomes de Oliveira

**Affiliations:** ^1^ Postgraduate Program in Dentistry, University of Pernambuco, Recife, State of Pernambuco, Brazil, ufpe.br

**Keywords:** biomarkers, cardiovascular diseases, periapical periodontitis, root canal therapy

## Abstract

**Background:**

Apical periodontitis (AP) is an inflammatory response to microbial infection of the root canal system. The microorganisms that cause AP can modulate the host’s immune response by secreting inflammatory biomarkers, which might be associated with cardiovascular diseases.

**Objective:**

To evaluate the existing evidence on the relationship between endodontic treatment of teeth with AP and changes in the levels of inflammatory biomarkers associated with cardiovascular risk.

**Materials and Methods:**

This systematic review was registered in PROSPERO (CRD42024574082) and followed the PRISMA guidelines (2020) and Cochrane Handbook (2023). Searches were performed in April 2025 in Medline/PubMed, Embase, Web of Science, Scopus, Cochrane, LILACS, and gray literature, with no publication year restrictions. The PICO strategy was followed: (1) population: healthy adult patients with AP; (2) intervention: evaluation of the levels of inflammatory biomarkers of cardiovascular disease risk (IL‐6, hs‐CRP, IL‐1β, TNF‐α, and others) before and after endodontic procedures; (3) comparison: the same patients before any endodontic intervention; and (4) outcomes: reduction in the levels of cardiovascular disease risk biomarkers.

**Results:**

Twenty clinical studies were included in the qualitative analysis, and 18 reported a reduction in inflammatory biomarkers after endodontic treatment. A meta‐analysis evaluated hs‐CRP at 1 and 6 months post‐treatment, as well as IL‐6 and TNF‐α at 6 months and 1 year post‐treatment and IL‐1β at 6 months. There was a significant reduction in hs‐CRP 6 months post‐treatment. No significant changes were found in the other biomarkers.

**Conclusion and Recommendations:**

A significant reduction in hs‐CRP was observed 6 months after endodontic treatment. However, there was no association between endodontic treatment of teeth with AP and the reduction in the other biomarkers evaluated in this systematic review and meta‐analysis. Furthermore, the certainty of evidence was classified as low according to the Grading of Recommendations Assessment, Development and Evaluation (GRADE) system, mainly because of the heterogeneity across primary studies and the lack of adequate control for potential confounding factors such as marginal periodontitis and smoking, which are linked to increased systemic inflammatory burden. Well‐designed clinical trials using rigorous control of these factors, standardized methodologies, and longer follow‐up periods are needed to confirm these findings.

## 1. Background

Apical periodontitis (AP) is an inflammatory disease that occurs around the tooth apex in response to microbial infection of the root canal system [1]. Given the proximity to periradicular tissues, bacteria located in the apical part of the root canal are the main causative agents of AP [2]. The development of AP is the result of an inflammatory response in periapical tissues and bone destruction, which depend on microbial colonization of the root canal, biofilm organization, and the degree of microbial virulence [3]. Microorganisms can cause direct tissue damage and modulate host immune responses by secreting products such as immunoglobulins, cytokines, chemokines, and other inflammatory markers [4].

At an individual level, it is estimated that half of the global population has at least one tooth with AP. This condition is one of the most prevalent diseases and the main cause of tooth loss [5–7]. However, AP can manifest in different ways, ranging from severe discomfort to a completely asymptomatic condition. The disease can remain unnoticed for years and can potentially interfere with the general health of affected individuals [8]. Studies have investigated the association between AP and systemic conditions such as metabolic syndrome, glycemic control, and cardiovascular diseases [9].

Cardiovascular diseases are a complex group of disorders and the leading cause of death worldwide, accounting for 30% of global mortality [10]. The literature has shown that chronic inflammatory conditions such as AP are related to the progression of atherosclerosis and acute cardiovascular events [11]. Signs of endothelial dysfunction have been detected in healthy young adults with AP [11, 12]. Although AP is a localized infection, microorganisms and their byproducts present in the root canal system can stimulate the systemic release of inflammatory mediators into the bloodstream, thereby promoting a persistent state of low‐grade systemic inflammation. This process has been associated with alterations in diverse inflammatory and oxidative stress biomarkers related to cardiovascular risk, such as high‐sensitivity C‐reactive protein (hs‐CRP), matrix metalloproteinase‐8 (MMP‐8), E‐selectin, interleukin 1 (IL‐1), IL‐2, IL‐6, and nitric oxide, which play an important role in endothelial dysfunction and atherosclerosis progression [4, 7].

Furthermore, a reduction in the levels of inflammatory biomarkers associated with cardiovascular diseases has been reported after endodontic treatment of teeth with AP [12–15]. Therefore, the aim of this systematic review with meta‐analysis was to evaluate the existing evidence on the relationship between endodontic treatment of teeth with AP and changes in the levels of inflammatory biomarkers of cardiovascular risk.

## 2. Materials and Methods

### 2.1. Protocol and Registration

This systematic review and meta‐analysis were registered in PROSPERO (CRD42024574082) and were conducted following the PRISMA guidelines [16] and Cochrane Handbook [17].

### 2.2. Research Question and Eligibility Criteria

The PICO framework was used: (1) population (P): healthy adult patients with AP; (2) intervention (I): evaluation of the levels of inflammatory biomarkers of cardiovascular disease risk (IL‐6, hs‐CRP, IL‐1β, tumor necrosis factor alpha (TNF‐α), and others) before and after nonsurgical and surgical endodontic procedures; (3) comparison (C): the same patients before any endodontic intervention; and (4) outcomes (O): reduction in the levels of biomarkers of cardiovascular disease risk.

Studies that met the following criteria were considered eligible for this systematic review: randomized clinical trials, nonrandomized clinical trials, and cohort studies that evaluated the levels of inflammatory biomarkers of cardiovascular disease risk before and after endodontic procedures in healthy patients with AP. Animal studies, *ex vivo* studies, case reports, letters to the editor, opinions, and reviews were excluded. There was no publication year restriction.

### 2.3. Information Sources, Search Strategy, Study Selection, and Data Extraction and Collection

Searches were conducted independently by two reviewers (C.V.V.L. and J.M.M.) in the Medline/PubMed, Embase, Web of Science, Scopus, Cochrane, and LILACS databases in April 2025. The search was performed applying English terms to the databases, including free‐text keywords, MeSH terms, and Emtree terms, combined by the Boolean operators “OR” or “AND” (the detailed search strategies for the databases are listed in Supplementary Table 1). Additional searches were performed in Google Scholar, ProQuest, and Dissertations & Theses Citation Index and through manual screening of the reference lists of the included studies.

The myendnoteWeb (Clarivate Analytics, PA, USA) and Rayyan (rayyan.qcri.org) reference management software were used for duplicate removal and selection of the studies. The articles were selected in two steps (screening of titles and abstracts and full‐text reading) by the same independent reviewers (C.V.V.L. and J.M.M.). In each step, disagreements were resolved by consensus or consultation of a third reviewer (N.G.O.). Data were extracted independently by C.V.V.L. and J.M.M. using a data form (Table 1). The interexaminer kappa agreement exceeded 90% for both screening and extraction.

**TABLE 1:** Descriptive summary of the clinical studies included in this systematic review.

Study	Study design	Population/follow‐up period	Diagnosis	Intervention	Sample	Inflammatory marker levels before and after endodontic treatment (mean ± standard deviation)	Conclusion
Al‐Abdulla et al. [9]	Observational longitudinal	Before intervention (*n* = 65) 1 year after intervention (*n* = 50) 2 years after intervention (*n* = 37)	Apical periodontitis	Nonsurgical root canal retreatment (*n* = 20) Periapical surgery (*n* = 17)	Serum	Before ADMA (pg/mL): 142,242.43 ± 118,478.1007 E‐selectin (pg/mL): 30,058.798 ± 10,842.33889 FGF‐23 (pg/mL): 612.055 ± 491.4918536 hs‐CRP (pg/mL): 145,278.085 ± 100,108.4038 ICAM‐1 (pg/mL): 506,912.6 ± 274,915.8467 IL‐1β (pg/mL): 18.1225 ± 9.206133782 IL‐6 (pg/mL): 17.0125 ± 11.27660672 IL‐8 (pg/mL): 20.905 ± 11.78248516 MMP‐2 (pg/mL): 20,472.0925 ± 6963.604546 MMP‐8 (pg/mL): 4597.885 ± 3137.483691 MMP‐9 (pg/mL): 21,193.905 ± 10,360.32212 Pentraxin 3 (pg/mL): 2862.2 ± 1850.187422 TNF‐α (pg/mL): 26.1025 ± 14.44848587 VCAM‐1 (pg/mL): 754,208.08 ± 316,551.7468 1 year ADMA (pg/mL): 2138.31 ± 769.8069175 E‐selectin (pg/mL): 26,106.778 ± 10,045.03904 FGF‐23 (pg/mL): 30.2825 ± 4.343991019 hs‐CRP (pg/mL): 67,580.783 ± 27,693.66489 ICAM‐1 (pg/mL): 560,909.91 ± 344,455.065 IL‐1β (pg/mL): 18.4225 ± 5.922003149 IL‐6 (pg/mL): 19.375 ± 13.90790352 IL‐8 (pg/mL): 21.25 ± 12.58 MMP‐2 (pg/mL): 30,543.318 ± 7885.048011 MMP‐8 (pg/mL): 3611.2675 ± 2051.475948 MMP‐9 (pg/mL): 23,133.433 ± 10,929.91603 Pentraxin 3 (pg/mL): 1246.9125 ± 688.5638099 TNF‐α (pg/mL): 11.5475 ± 3.318728901 VCAM‐1 (pg/mL): 570,856.18 ± 174,249.7874 2 years ADMA (pg/mL): 1183.2075 ± 542.76358 E‐selectin (pg/mL): 42,409.158 ± 19,131.296 FGF‐23 (pg/mL): 68.14 ± 21.243515 hs‐CRP (pg/mL): 33,504.76 ± 17,094.363 ICAM‐1 (pg/mL): 935,300.31 ± 695,566.11 IL‐1β (pg/mL): 50.445 ± 12.346176 IL‐6 (pg/mL): 24.9625 ± 6.2224915 IL‐8 (pg/mL): 76.21 ± 50.753887 MMP‐2 (pg/mL): 7274.1375 ± 2199.212 MMP‐8 (pg/mL): 9250.735 ± 5091.9177 MMP‐9 (pg/mL): 23,724.415 ± 5278.743 Pentraxin 3 (pg/mL): 3260.3125 ± 1418.4058 TNF‐α (pg/mL): 33.5275 ± 11.148729 VCAM‐1 (pg/mL): 1,837,769.6 ± 797,722.82	After 2 years, serum MMP‐2 levels were higher in the healing group compared to the healed group. In contrast, serum levels of hs‐CRP, ADMA, and MMP‐2 were significantly reduced. There was a significant increase in all other biomarkers, except for FGF‐23 and ICAM‐1.

Bains et al. [22]	Observational longitudinal	Before intervention (*n* = 15) 1 month after intervention (*n* = 11)	Lesions of endodontic origin	Primary root canal treatment (*n* = 15)	Serum	Before hs‐CRP (mg/L): 6.18 ± 3.72 1 month hs‐CRP (mg/L): 3.92 ± 3.59	Root canal treatment reduced the serum levels of hs‐CRP in patients with lesions of endodontic origin.

Bakhsh et al. [14]	Observational longitudinal	Before intervention (*n* = 65) 3 months after intervention (*n* = 40) 6 months after intervention (*n* = 37) 1 year after intervention (*n* = 50)	Apical periodontitis	Nonsurgical root canal retreatment (*n* = 35) Periapical surgery (*n* = 30)	Serum	Before ADMA (pg/mL): 39,284.02 ± 131,064.5 C3 (pg/mL): 909.51 ± 642.48 E‐selectin (pg/mL): 26,154.19 ± 13,726.45 FGF‐23 (pg/mL): 172.17 ± 542.78 hs‐CRP (pg/mL): 78,287.56 ± 87,056.44 ICAM‐1 (pg/mL): 408,464.5 ± 283,934.05 IL‐1β (pg/mL): 17.53 ± 14.32 IL‐6 (pg/mL): 7.06 ± 7.93 IL‐8 (pg/mL): 18.35 ± 24.21 MMP‐2 (pg/mL): 16,724.39 ± 6487.86 MMP‐8 (pg/mL): 2752.02 ± 2425.22 MMP‐9 (pg/mL): 16,647.96 ± 11,737.83 Pentraxin 3 (pg/mL): 1843.61 ± 1671.74 TNF‐α (pg/mL): 18.37 ± 22.66 VCAM‐1 (pg/mL): 705,054.43 ± 289,750.99 3 months ADMA (pg/mL): 2610 ± 2029 C3 (pg/mL): 1832 ± 654.4 E‐selectin (pg/mL): 28,335 ± 11,929 FGF‐23 (pg/mL): 36.2 ± 13.33 hs‐CRP (pg/mL): 180,677 ± 142,781 ICAM‐1 (pg/mL): 412,472 ± 255,324 IL‐1β (pg/mL): 22.21 ± 9.04 IL‐6 (pg/mL): 5.88 ± 5.80 IL‐8 (pg/mL): 22.59 ± 35.15 MMP‐2 (pg/mL): 24,466 ± 21,834 MMP‐8 (pg/mL): 2907 ± 2778 MMP‐9 (pg/mL): 23,374 ± 16,122 Pentraxin 3 (pg/mL): 1032 ± 955.7 TNF‐α (pg/mL): 8.96 ± 3.75 VCAM‐1 (pg/mL): 599,187 ± 180,750 6 months ADMA (pg/mL): 6312 ± 1268 C3 (pg/mL): 970 ± 666.2 E‐selectin (pg/mL): 26,962 ± 10,582 FGF‐23 (pg/mL): 35.14 ± 13.43 hs‐CRP (pg/mL): 120,109 ± 71,764 ICAM‐1 (pg/mL): 385,795 ± 272,567 IL‐1β (pg/mL): 25.4 ± 10.48 IL‐6 (pg/mL): 8.73 ± 24.01 IL‐8 (pg/mL): 20.03 ± 11.17 MMP‐2 (pg/mL): 14,008 ± 1303 MMP‐8 (pg/mL): 3138 ± 2362 MMP‐9 (pg/mL): 14,449 ± 8278 Pentraxin 3 (pg/mL): 1220 ± 868.3 TNF‐α (pg/mL): 10.18 ± 3.06 VCAM‐1 (pg/mL): 656,742 ± 202,390 1 year ADMA (pg/mL): 1872 ± 600.9 C3 (pg/mL): 922.9 ± 286.8 E‐selectin (pg/mL): 22,849 ± 9650 FGF‐23 (pg/mL): 27.04 ± 7.62 hs‐CRP (pg/mL): 65,825 ± 31,201 ICAM‐1 (pg/mL): 433,727 ± 335,354 IL‐1β (pg/mL): 16.16 ± 7.04 IL‐6 (pg/mL): 6.65 ± 8.93 IL‐8 (pg/mL): 14.21 ± 7.21 MMP‐2 (pg/mL): 29,649 ± 11,388 MMP‐8 (pg/mL): 2580 ± 1797 MMP‐9 (pg/mL): 18,877 ± 11,371 Pentraxin 3 (pg/mL): 978.3 ± 791.5 TNF‐α (pg/mL): 11.18 ± 3.47 VCAM‐1 (pg/mL): 553,902 ± 180,482	After 3 months of treatment, there was an increase in serum levels of IL‐1β, IL‐8, hs‐CRP, C3, MMP‐2, and MMP‐9. At 6 months of follow‐up, IL‐1β and IL‐8 continued to increase, and there was a decrease in FGF‐23, hs‐CRP, C3, MMP‐2, and MMP‐9. After 1 year of treatment, FGF‐23, pentraxin‐3, and ADMA were reduced.

Bakhsh et al. [30]	Observational longitudinal	Before intervention (*n* = 65) 1 year after intervention (*n* = 50) 2 years after intervention (*n* = 37)	Apical periodontitis	Nonsurgical root canal retreatment (*n* = 35) Periapical surgery (*n* = 30)	Saliva	Before E‐selectin (pg/mL): 159.32 ± 47.6507 FGF‐23 (pg/mL): 13.68 ± 2.5784859 hs‐CRP (pg/mL): 558.825 ± 247.0479 ICAM‐1 (pg/mL): 19,165.975 ± 5684.1953 IL‐1β (pg/mL): 436.605 ± 126.7471 IL‐6 (pg/mL): 21.9125 ± 4.1430 IL‐8 (pg/mL): 671.62 ± 305.17063 MMP‐2 (pg/mL): 786.405 ± 291.5097 MMP‐8 (pg/mL): 93,412.89 ± 41,382.137 MMP‐9 (pg/mL): 13,002.245 ± 6722.2066 Pentraxin 3 (pg/mL): 8632.25 ± 5927.742668 TNF‐α (pg/mL): 24.7925 ± 7.186889 VCAM‐1 (pg/mL): 4390.59 ± 1777.8319 **1 year** NI 2 years NI	Higher levels of salivary cytokines, MMPs, and vascular adhesion molecules in post‐treatment assessments are related to periapical bone healing and remodeling, while salivary FGF‐23 and hs‐CRP may be prognostic biomarkers.

Bergandi et al. [12]	Observational longitudinal	Before intervention (*n* = 23) 2 months after intervention (*n* = NI) 1 year after intervention (*n* = 21)	Chronic apical periodontitis	Primary root canal treatment (*n* = 23)	Plasma	Before Endothelin‐1 (pg/mL): 1.65 ± 0.41 E‐selectin (ng/mL): 31.71 ± 4.78 ICAM‐1 (pg/mL): 238.74 ± 63.31 IL‐1(pg/mL): 9.59 ± 1.03 IL‐6 (pg/mL): 7.63 ± 3.04 sCD14 (mg/mL): 6.09 ± 1.77 VCAM‐1 (ng/mL): 533.47 ± 84.55 TNF‐a (pg/mL): 4.62 ± 0.89 2 months Endothelin‐1 (pg/mL): 1.23 ± 0.31 E‐selectin (ng/mL): 28.21 ± 4.54 ICAM‐1 (pg/mL): 187.05 ± 61.69 IL‐1 (pg/mL): 7.84 ± 0.88 IL‐6 (pg/mL): 7.99 ± 5.36 sCD14 (mg/mL): 3.85 ± 1.51 sVCAM‐1 (ng/mL): 535.46 ± 74.86 TNF‐a (pg/mL): 4.64 ± 0.88 1 year Endothelin‐1 (pg/mL): 1.11 ± 0.21 E‐selectin (ng/mL): 27.45 ± 4.62 ICAM‐1 (pg/mL): 181.59 ± 64.17 IL‐1 (pg/mL): 7.77 ± 0.87 IL‐6 (pg/mL): 6.84 ± 4.38 sCD14 (mg/mL): 3.77 ± 1.57 sVCAM‐1 (ng/mL): 490.88 ± 106.07 TNF‐a (pg/mL): 4.51 ± 0.85	Concentrations of endothelin‐1, ICAM‐1, sCD14, and E‐selectin were significantly reduced at 2 and 12 months post‐treatment.

Brosco et al. [23]	Observational longitudinal	Before intervention (*n* = 13) 1 month after intervention (*n* = 13)	Chronic periapical lesions	Primary root canal treatment (*n* = 11) Periapical surgery (*n* = 1) Extraction (*n* = 1)	Plasma	Before hs‐CRP (mg/L): 2.42 ± 2.0 1 month hs‐CRP (mg/L): 2.5 ± 2.6	There was no difference in serum hs‐CRP levels after 1 month of treatment of teeth with AP.

Dezerega et al. [24]	Observational longitudinal	Before intervention (*n* = 16) 1 week after intervention (*n* = 16)	Asymptomatic apical periodontitis	Primary root canal treatment (*n* = 16)	Gingival crevicular fluid	Before MMP‐2: NI MMP‐9: NI 1 week NI	Apical lesions exhibit an oxidative imbalance along with increased MMP‐2 and MMP‐9. This oxidative imbalance can be demonstrated in gingival crevicular fluid of teeth with AP and is restored to normal levels after conservative endodontic treatment.

Garrido et al. [7]	Observational longitudinal	Before intervention (*n* = 29) 1 month after intervention (*n* = 26) 6 months after intervention (*n* = 19)	Primary apical periodontitis	Primary root canal treatment (*n* = 26)	Serum	Before E‐selectin (pg/mL): 28,512.6 ± 9382.9 hs‐CRP (mg/L): 2.8 ± 3.3 IL‐1β. (pg/mL): 17.5 ± 3.4 IL‐6 (pg/mL): 7.3 ± 1.1 IL‐10 (pg/mL): 6.9 ± 0.8 TNF‐α (pg/mL): 16.7 ± 3.0 1 month E‐selectin (pg/mL): 28,033.9 ± 7089.9 hs‐CRP (mg/L): 1.8 ± 2.4 IL‐1β. (pg/mL): 17.3 ± 2.8 IL‐6 (pg/mL): 7.1 ± 0.8 IL‐10 (pg/mL): 6.8 ± 0.6 TNF‐α (pg/mL): 16.9 ± 2.9 6 months E‐selectin (pg/mL): 29,260.6 ± 12,460.9 hs‐CRP (mg/L): 2.0 ± 2.1 IL‐1β (pg/mL): 17.3 ± 2.9 IL‐6 (pg/mL): 7.4 ± 1.4 IL‐10 (pg/mL): 7.4 ± 2.3 TNF‐α (pg/mL): 17.8 ± 4.3	Hs‐CRP and mCRP are reduced after root canal treatment in individuals with AP at cardiovascular risk.

Gandhe et al. [21]	Randomized controlled trial	Group 1 (foraminal enlargement): Before intervention (*n* = 30) 3 days after intervention (*n* = 30) Group 2 (no foraminal enlargement): Before intervention (*n* = 30) 3 days after intervention (*n* = 30)	Asymptomatic apical periodontitis	Primary root canal treatment (Groups 1 and 2)	Serum	Before Group 1 ESR (mm/h): 21.67 ± 0.5 hs‐CRP (mg/L): 1.92 ± 0.5 Group 2 ESR (mm/h): 20.41 ± 0.5 hs‐CRP (mg/L): 1.70 ± 0.5 3 days Group 1 ESR (mm/h): 14.13 ± 0.5 hs‐CRP (mg/L): 1.15 ± 0.5 Group 2: ESR (mm/h): 20.36 ± 0.5 hs‐CRP (mg/L): 1.67 ± 0.5	Root canal treatment decreased serum CRP and ESR levels in systemically healthy individuals with AP with or without apical enlargement.

Giuggia et al. [13]	Observational longitudinal	Before intervention (*n* = 23) 1 year after intervention (*n* = 19)	Apical periodontitis	Primary root canal Treatment	Plasma	Before Endothelin‐1 (pg/mL): 1.65 ± 0.1 E‐selectin (ng/mL): 31.71 ± 1.1 ICAM‐I (pg/mL): 238.7 ± 14.5 IL‐I (pg/mL): 9.59 ± 0.24. CD14 (μg/mL): 6.09 ± 1.77 VCAM‐I (ng/mL): 538 ± 19.8 1 year Endothelin‐1 (pg/mL): 1.11 ± 0.02 E‐selectin (ng/mL): 27.45 ± 1.1 ICAM‐I (pg/mL): 181.6 ± 15.6 IL‐I (pg/mL): 7.77 ± 0.2 sCD14 (μg/mL): 3.75 ± 1.49 sVCAM‐I (ng/mL): 491 ± 25.7.	There was a decrease in endothelin‐1, E‐selectin, CAM‐I, IL‐1, and CD14 after endodontic treatment.

Hepsenoglu et al. [15]	Randomized controlled trial	Group 1 (SWEEPS mode of the Er: YAG laser): Before intervention (*n* = 15) 3 days after intervention (*n* = 15) Group 2 (Passive ultrasonic irrigation): Before intervention (*n* = 15) 3 days after intervention (*n* = NI)	Chronic apical periodontitis	Primary root canal treatment (Groups 1 and 2)	Serum	Before IL‐1β (Group 1) (mg/L): 53 ± 18 IL‐1β (Group 2) (mg/L): 60.7 ± 21.6 3 days IL‐1β (Group 1) (mg/L): 33.1 ± 9.1 IL‐1β (Group 2) (mg/L): 31.0 ± 13.0	SWEEPS activation and PUI were equivalent in terms of reducing inflammation detected by IL‐1β.

Inchingolo et al. [25]	Observational longitudinal	Before intervention (*n* = 33) 1 month (*n* = NI) 3 months (*n* = NI)	Chronic apical periodontitis	NI	Plasma	Before BAP (μM/L): 1790 ± 64 ROM (U CARR): 458 ± 36 1 month BAP (μM/L): 1847 ± 65 ROM (U CARR): 311 ± 30 3 months BAP (μM/L): 2365 ± 110 ROM (U CARR): 274 ± 14	After 1 and 3 months, an increase in BAP levels and a decrease in ROM following endodontic treatment were observed.
Kumar et al. [33]	Observational longitudinal	Before intervention (*n* = 25) 6 months after intervention (*n* = 22)	Asymptomatic apical periodontitis	Primary root canal treatment	Serum	Before hs‐CRP (mg/L): 3.37 ± 2.69 6 months hs‐CRP (mg/L): 1.79 ± 1.65	A reduction in hs‐CRP was observed after endodontic treatment.

Marton et al. [31]	Observational longitudinal	Before intervention (*n* = 36) 1 week after intervention (*n* = NI) 3 months after intervention (*n* = NI)	Chronic periapical granuloma	Primary endodontic with periapical surgery (*n* = 36)	Serum	Before AAT (g/L): 3.02 ± 1.01 AMG (g/L): 2.80 ± 1.01 C3 (g/L): 1.65 ± 0.39 CER (IU/L): 184 ± 27 hs‐CRP (mg/L): 6.58 ± 4.15 HPT (g/L): 0.99 ± 0.26 1 week AAT (g/L): 2.26 ± 1.00 AMG (g/L): 1.78 ± 1.13 C3 (g/L): 1.53 ± 0.44 CER (IU/L): 143 ± 45 hs‐CRP (mg/L): 6.44 ± 6.70 HPT (g/L): 0.89 ± 0.39 3 months AAT (g/L): 2.13 ± 0.73 AMG (g/L): 1.81 ± 0.70 C3 (g/L): 1.38 ± 0.43 CER (IU/L): 140 ± 32 hs‐CRP (mg/L): 3.90 ± 1.84 HPT (g/L): 0.68 ± 0.37	After 7 days, there was a decrease in serum levels of AAT and CER. After 3 months, there was a decrease in all proteins.

Marton and Kiss [32]	Observational longitudinal	Before intervention (*n* = 36) 1 week after intervention (*n* = NI) 3 months after intervention (*n* = NI)	Chronic periapical lesions	Primary endodontic with periapical surgery (*n* = 36)	Serum	Before AAT (g/L): 3.0 ± 1.0 AMG (g/L): 2.8 ± 1.0 C3 (g/L): 1.7 ± 0.4 CER (IU/L): 80 ± 30 hs‐CRP (mg/L): 6.6 ± 4.2 HPT (g/L): 1.0 ± 0.3 IgA (g/L): 2.1 ± 0.6 IgG (g/L): 12.3 ± 3.0 IgM (g/L) 1.9 ± 0.9. 1 week AAT (g/L): 2.3 ± 1.0 AMG (g/L): 1.8 ± 1.1 C3 (g/L): 1.5 ± 0.4 CER (IU/L): 140 ± 50 hs‐CRP (mg/L): 6.4 ± 6.7 HPT (g/L): 0.9 ± 0.4 IgA (g/L): 2.2 ± 0.6 IgG (g/L): 11.9 ± 2.7 IgM (g/L): 1.9 ± 0.8 3 months AAT (g/L): 2.1 ± 0.7 AMG (g/L): 1.8 ± 0.7 C3 (g/L): 1.4 ± 0.4 CER (IU/L): 140 ± 30 hs‐CRP (mg/L): 3.9 ± 1.8 HPT (g/L): 0.7 ± 0.4 IgA (g/L): 2.2 ± 0.6 IgG (g/L): 11.8 ± 3.0 IgM (g/L): 2.1 ± 0.7	Serum levels of AAT, AMG, and CER decreased 7 days after endodontic treatment. After 3 months of treatment, there was a decrease in the serum levels of all proteins.

Multari et al. [26]	Observational longitudinal	Before intervention (*n* = 27) 6 months after intervention (*n* = 24) 1 year after intervention (*n* = 24)	Chronic apical periodontitis	Primary root canal treatment (*n* = 27)	Plasma	Before IL‐1β (pg/mL): 11.90 ± 2.518 IL‐6 (pg/mL): 13.70 ± 3.137 IL‐8 (pg/mL): 87.20 ± 21.92 TNF‐α (pg/mL): 4.082 ± 0.9567 6 months IL‐1β (pg/mL): 9.799 ± 2.950 IL‐6 (pg/mL): 9.811 ± 3.562 IL‐8 (pg/mL): 65.32 ± 21.30 TNF‐α (pg/mL): 3.506 ± 1.469 1 year IL‐1β (pg/mL): 8.652 ± 2.647 IL‐6 (pg/mL): 8.675 ± 3.955 IL‐8 (pg/mL): 55.21 ± 19.41 TNF‐α (pg/mL): 3.579 ± 1.325	Serum levels of IL‐1β, IL‐6, and IL‐8 decreased after 6 months of treatment and were further reduced after 12 months.

Palafox Sanchez et al. [27]	Observational longitudinal	Before intervention (*n* = 30) 2 weeks after intervention (*n* = 18)	Acute apical abscesses and trismus onset	Antibiotic therapy combined with primary root canal treatment (*n* = 18)	Serum	Before IFN‐γ: NI TNF‐α: NI IL‐1β: NI IL‐4: NI IL‐6: NI IL‐17: NI IL‐10: NI 2 weeks IFN‐γ: NI TNF‐α: NI IL‐1β: NI IL‐4: NI IL‐6: NI IL‐17: NI IL‐10: NI	TNF‐α levels decreased after endodontic treatment.

Poornima et al. [34]	Observational longitudinal	Before intervention (*n* = 15) 6 months after intervention (*n* = 15)	Chronic apical periodontitis	Primary root canal treatment (*n* = 15)	Serum	Before hs‐CRP (mg/L): 2.88 ± 1.06 6 months hs‐CRP (mg/L): 1.34 ± 0.52	Root canal treatment can reduce serum hs‐CRP levels in systemically healthy individuals with AP.

Sathyanarayanan et al. [28]	Observational longitudinal	Before intervention (*n* = 25) 1 week after intervention (*n* = NI)	Asymptomatic apical periodontitis	Primary root canal Treatment	Blood samples	Before Leuk (mm3): 8966.4 ± 1337.07 Lymph (cu.mm): 28.84 ± 4.853 Eos (cu.mm): 4.8 ± 1.384 1 week Leuk (mm3): 7918 ± 783.19 Lymph (cu.mm): 24.96 ± 2.684 Eos (cu.mm): 2.52 ± 0.7141	Complete healing of asymptomatic AP lesions results in an overall reduction in systemic inflammatory cells.

Yun et al. [29]	Observational longitudinal	Before intervention (*n* = 11) 1 month after intervention (*n* = NI) 6 months after intervention (*n* = NI)	Asymptomatic or symptomatic apical periodontitis	Primary root canal Treatment	Gingival crevicular fluid	Before IL‐1β: NI IL‐4: NI IL‐13: NI MMP‐2: NI MMP‐9: NI 1 month IL‐1β: NI IL‐4: NI IL‐13: NI MMP‐2: NI MMP‐9: NI 6 months IL‐1β: NI IL‐4: NI IL‐13: NI MMP‐2: NI MMP‐9: NI	Expression of IL‐1β, IL‐4, and MMP‐9 was reduced after successful endodontic treatment.

*Note:* AAT, alpha‐1 antitrypsin; ADMA, asymmetric dimethylarginine; AMG, alpha‐2 macroglobulin; C3, complement component 3; CER, ceruloplasmin; Eos, eosinophil count; ET‐1, endothelin‐1; HPT, haptoglobin; ICAM‐1, intercellular adhesion molecule 1; Ig, immunoglobulin; IL, interleukin; Leuk, leukocyte count; Lymph, lymphocyte count; MMP, matrix metalloproteinase; sCD14, soluble CD14.

Abbreviations: BAP, biological antioxidant potential; ESR, erythrocyte sedimentation rate; FGF‐23, fibroblast growth factor 23; hs‐CRP, high‐sensitivity C‐reactive protein; NI: not informed; ROM, reactive oxygen metabolites; TNF‐α, tumor necrosis factor alpha; VCAM‐1, vascular cell adhesion molecule 1.

### 2.4. Risk of Bias

The risk of bias was assessed independently by two reviewers (C.V.V.L. and J.M.M.) following pre‐established criteria. Randomized controlled trials were evaluated using the Revised Cochrane Risk‐of‐Bias 2 (RoB 2) tool [18]. Observational‐longitudinal studies were assessed using the Newcastle–Ottawa Scale (NOS), which assigns a maximum of 8 points in three domains: participant selection, comparability, and ascertainment of outcomes of interest [19]. Study quality was classified as *good* (score of 8 or 7 points), *fair* (score of 6 or 5 points), or *poor* (score less than 5 points).

### 2.5. Data Synthesis and Analysis

Descriptive/qualitative data were obtained for each outcome. In addition, meta‐analysis was performed by inverse variance weighting of the mean difference between the outcomes assessed before and after the intervention (endodontic treatment). A random‐effects model was used because of the presence of heterogeneity, considering a 95% confidence interval (CI) and significance at *p* < 0.05. The Reviewer Manager 5 program (Cochrane Group) was used for meta‐analysis and the creation of forest plots.

### 2.6. Certainty of Evidence

The certainty of evidence was rated using the Grading of Recommendations Assessment, Development, and Evaluation (GRADE) methodology [20]. Two reviewers (C.V.V.L. and J.M.M.) independently performed the assessment (Supplementary Table 2). Disagreements were resolved through discussions or consultation with a third reviewer (N.G.O.).

## 3. Results

### 3.1. Study Selection

**FIGURE 1:**
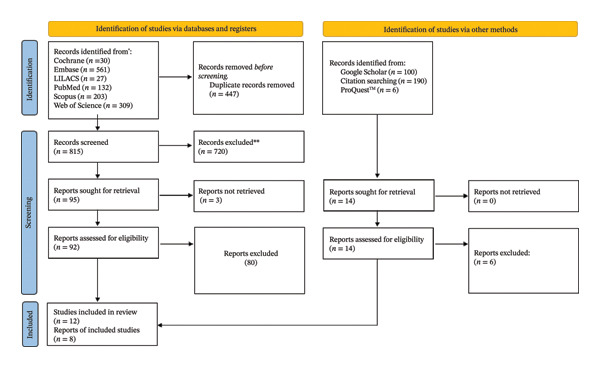
PRISMA flow diagram of the study selection process. ^∗^Total number of records identified in each database. ^∗∗^Records excluded manually.

The database searches retrieved 1262 articles. After duplicate removal and thorough screening of titles and abstracts, 92 articles were selected for full‐text reading. Of these, 80 articles were excluded because they did not meet the inclusion criteria; thus, 12 studies were selected for this review. Eight articles were retrieved by additional searches, 2 from the gray literature, 4 from Google Scholar, and 2 by screening the references of the included articles (Figure 1). All 20 included articles were interventional studies published between 1988 and 2024; two were randomized controlled trials [15, 21] and 18 were observational‐longitudinal studies [7, 12, 13, 22–29]. The studies by Al‐Abdula et al. [9], Bakhsh et al. [14], and Bakhsh et al. [30] were identified as the same clinical study. For meta‐analysis, only the data extracted from the study by Bakhsh et al. [14] were used because of the compatibility of the follow‐up with the other studies included in that analysis. The articles by Bergandi et al. [12] and Giuggia et al. [13] were also identified as the same clinical study. The study by Bergandi et al. [12] was used for data analysis because it included a larger sample and more biomarkers. The same also applied to the studies by Marton et al. [31] and Marton et al. [32]. The latter study was chosen for data analysis because it evaluated more biomarkers.

All 20 articles were written in English, and 13 were published over the last 5 years [7, 9, 14, 15, 21, 22, 26–29, 33, 34]. Six authors [9, 14, 24, 26, 27, 29] were contacted to request missing data. However, only Multari et al. [26] provided the requested information. All included studies were submitted to qualitative analysis (Table 1). For quantitative analysis, six studies [7, 12, 22, 26, 33, 34] that permitted the extraction of means and standard deviations and had compatible measurement units and follow‐ups were included in the meta‐analysis (Figure 2).

FIGURE 2Comparison of follow‐up levels of (a) high‐sensitivity C‐reactive protein (Hs‐CRP) at 1 month, (b) hs‐CRP at 6 months, (c) interleukin 6 (IL‐6) at 6 months, (d) IL‐6 at 1 year, (e) tumor necrosis factor alpha (TNF‐α) at 6 months, (f) TNF‐α at 1 year, and (g) interleukin 1 beta (IL‐1β) at 6 months.

**Figure:**
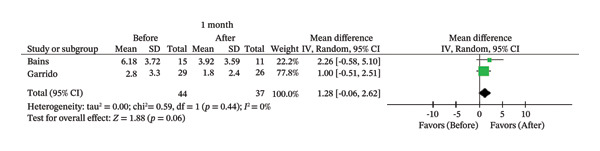
(a)

**Figure:**
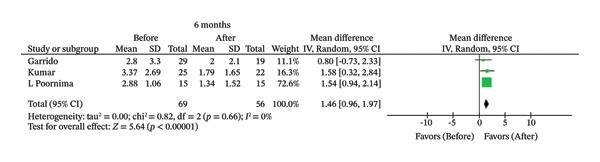
(b)

**Figure:**
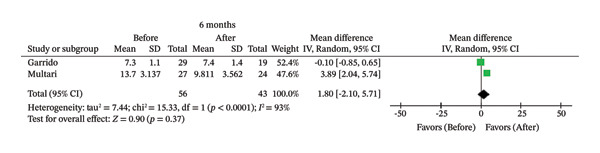
(c)

**Figure:**
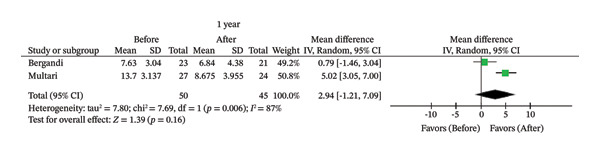
(d)

**Figure:**
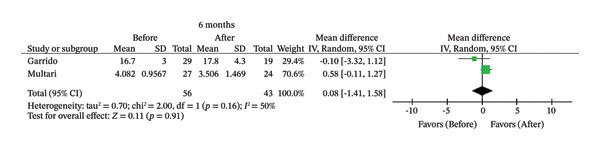
(e)

**Figure:**
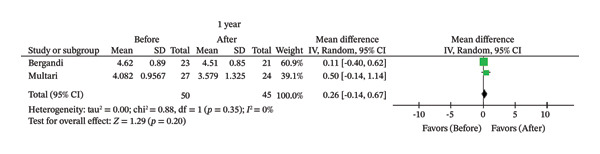
(f)

**Figure:**
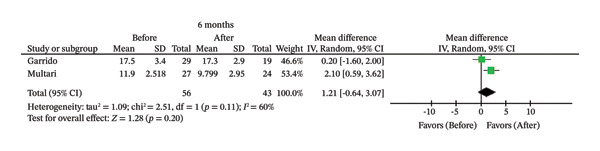
(g)

### 3.2. Characteristics of the Included Studies

The 20 included studies evaluated the concentration of biomarkers before and after endodontic treatment. However, substantial variability was observed across studies regarding the type of endodontic infections included, diagnostic criteria of AP, biomarkers assessed, biomarker measurement methods, and time intervals adopted for post‐treatment evaluation. Seventeen articles used peripheral blood samples for this evaluation, with serum in 11 [7, 9, 14, 15, 21, 22, 27, 31–34] and plasma in five [12, 13, 23, 25, 26]; one did not specify the type of sample [28]. Two other studies used gingival crevicular fluid samples [24, 29], and one used a saliva sample [30]. Although all 20 studies measured biomarkers related to cardiovascular risk, only 10 explicitly focused on cardiovascular risk. The follow‐up period ranged from 3 days to 2 years.

The treatments consisted of primary endodontic treatment [7, 12, 13, 15, 21–24, 26–29, 33, 34], nonsurgical endodontic retreatment [9, 14, 30], periapical surgery [9, 14, 23, 30], and endodontic retreatment combined with periapical surgery [31, 32]. In the study by Palafox‐Sanchez et al. [27], antibiotic therapy was administered prior to primary endodontic treatment, and inflammatory biomarkers were measured at three time points: (1) before any intervention; (2) after antibiotic therapy and before endodontic treatment; and (3) after endodontic treatment. For the purposes of this systematic review, only the data collected after the completion of endodontic treatment were considered. In the study by Brosco et al. [23], one patient underwent extraction of a previously endodontically treated tooth. Although the procedure was not in line with the objectives of this review, the patient could not be excluded from the analysis of the results. The article also did not report any exclusion criteria but met the inclusion criteria established for the review.

### 3.3. Characteristics of the Study Population and Main Findings

This systematic review included a total of 642 patients. However, sex or age distribution could not be determined because of incomplete reporting in the primary studies. Most of the included articles addressed chronic manifestations of AP (Table 1). Inflammatory biomarkers of cardiovascular risk were investigated in all studies. The most frequently evaluated inflammatory biomarkers were hs‐CRP, IL‐1β, and IL‐6 (Table 1).

### 3.4. Meta‐Analysis of Inflammatory Biomarkers: Patients With AP Before vs. After Treatment According to Follow‐Up Period

Although the studies included in this systematic review evaluated the serum levels of various biomarkers before and after endodontic treatment, meta‐analysis was only possible for hs‐CRP, IL‐6, IL‐1β, and TNF‐α. This limitation was due to the fact that the meta‐analyses were stratified according to follow‐up period, and only these biomarkers were consistently evaluated at comparable time points across studies considering the same type of infection (primary or secondary/persistent) and the same type of intervention (primary endodontic treatment or endodontic retreatment). Since Bergandi et al. [12] and Giuggia et al. [13] analyzed the same cohort, only one dataset from the overlapping cohort was included to avoid duplication and overestimation of the results.

The corresponding forest plots are displayed in Figure 2. Of the 11 studies that evaluated hs‐CRP [7, 9, 14, 21–23, 26, 31–34], only two with 1‐month follow‐up [7, 22] and three with 6‐month follow‐up [7, 33, 34] were included in the meta‐analysis. No statistically significant difference was found in hs‐CRP levels before and 1 month after endodontic treatment (MD = 1.28; 95% CI [−0.06, 2.62]; *p* = 0.06; *I* ^2^ = 0%). However, a statistically significant reduction in hs‐CRP was observed 6 months after treatment (MD = 1.46; 95% CI [0.96, 1.97]; *p* < 0.00001; *I* ^2^ = 0%).

Seven studies evaluated IL‐6 and TNF‐α [7, 9, 12, 14, 26, 27, 30]; however, there were only two studies with 6‐month follow‐up [7, 26] and two with 1‐year follow‐up [12, 26] that assessed these biomarkers using the same type of intervention (primary endodontic treatment or endodontic retreatment). No significant differences in IL‐6 were observed after 1 year (MD = 2.94; 95% CI [−1.21, 7.09]; *p* = 0.16; *I* ^2^ = 87%) or 6 months (MD = 1.80; 95% CI [−2.10, 5.71]; *p* = 0.37; *I* ^2^ = 93%). Similarly, there were no statistically significant changes in TNF‐α after 1 year (MD = 0.26; 95% CI [‐0.14, 0.67]; *p* = 0.20; *I* ^2^ = 0%) or 6 months (MD = 0.08; 95% CI [−1.41, 1.58]; *p* = 0.91; *I* ^2^ = 50%). Eight studies evaluated IL‐1β [7, 9, 14, 15, 26, 27, 29, 30]. However, only two studies [7, 26] provided 6‐month follow‐up data. Analysis of these studies revealed no statistically significant difference between pre‐ and post‐treatment levels (MD = 1.21; 95% CI [−0.64, 3.07]; *p* = 0.20; *I* ^2^ = 60%).

### 3.5. Risk of Bias Assessment

The RoB 2 tool (Figure 3) was used for two studies [15, 21], both classified as having a moderate risk of bias. This classification was primarily due to the short follow‐up period of 3 days, which is insufficient to confirm the resolution of AP. Additionally, the study by Gandhe et al. [21] did not describe the randomization process. Eighteen studies [7, 9, 12–14, 22–34] were evaluated using the NOS (Figure 3), and 10 of them were classified as poor. Regarding the second domain (comparability), a star was not assigned when information on general health or oral health status of the control participants was absent or incompletely reported. Studies that did not receive this star were classified as poor [12, 22, 23, 25, 27, 28, 31–34]. Regarding the third domain (outcome), for the item “Was follow‐up long enough for outcomes to occur?” A minimum follow‐up period of 6 months was considered adequate by the authors. Studies with shorter follow‐up did not receive a star [22–25, 27, 28, 31, 32]. Additionally, in the same domain, for the item “Adequacy of follow‐up of cohorts,” studies reporting a loss to follow‐up greater than 30% did not receive a star [7, 9, 12, 29–32].

FIGURE 3Risk of bias assessment. (a) Randomized trials assessed using the RoB 2 tool. (b) Nonrandomized trials assessed using the NOS.

**Figure:**
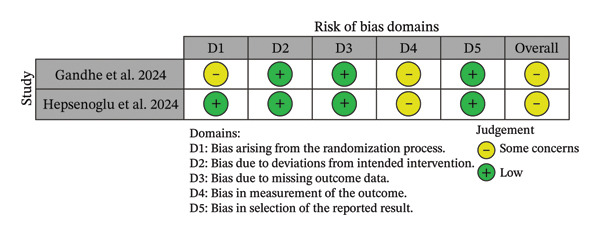
(a)

**Figure:**
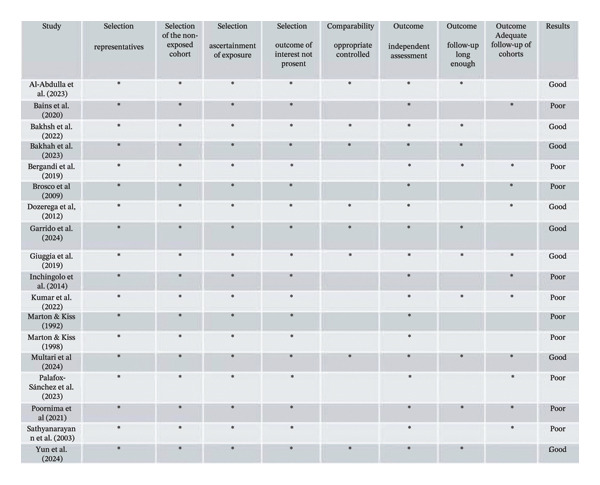
(b)

## 4. Discussion

Recent research has increasingly focused on the relationship between inflammatory diseases and cardiovascular conditions [35]. However, the specific effect of endodontic treatment for AP on biomarkers of cardiovascular risk remains undefined. Previous systematic reviews reported that the presence of AP is associated with an increase in inflammatory biomarkers in systemic circulation, gingival crevicular fluid, and saliva [4, 36, 37]. Another systematic review with meta‐analysis evaluated the impact of endodontic treatment on systemic inflammatory mediators but did not focus specifically on patients with AP or biomarkers of cardiovascular risk [6]. This systematic review was conducted to address this gap. We also performed a meta‐analysis to compare the levels of IL‐6, IL‐1β, hs‐CRP, and TNF‐α analyzed by the studies before and after endodontic treatment of patients with AP.

Interleukins are proinflammatory cytokines associated with cardiovascular diseases [36, 38, 39]. IL‐1β is a proatherogenic cytokine that plays an important role in both local inflammatory responses and systemic inflammatory processes. In AP, IL‐1β contributes to the progression of periapical inflammation by promoting the activation of bone resorption and inducing the production of proteinases involved in tissue degradation. Its biological effects are dependent on the concentrations present in tissues and systemic circulation. Thus, elevated IL‐1β levels such as those observed in AP may not only contribute to local tissue destruction but also promote systemic inflammatory responses related to endothelial dysfunction and atherosclerosis progression, potentially increasing cardiovascular risk [40]. However, studies on the effects of endodontic treatment of teeth with AP on serum IL‐1β levels have reported conflicting results. Garrido et al. [7] observed no changes at 6 months, while Multari et al. [26] reported a reduction. The present meta‐analysis did not reveal a significant reduction in IL‐1β after 6 months (*p* = 0.20), suggesting that longer periods may be necessary to identify systemic changes in this biomarker. It is important to highlight that invasive dental procedures such as endodontic treatment can induce transient elevations in the levels of systemic inflammatory markers, which may temporarily increase the risk of vascular events in susceptible individuals [41]. Nevertheless, there is evidence suggesting that successful endodontic treatment can result in a long‐term sustained reduction in inflammatory mediators and markers of endothelial dysfunction, compensating for the short‐term adverse inflammatory effects associated with the procedure [14]. Although Garrido et al. [7] and Multari et al. [26] investigated only primary endodontic infections, this meta‐analysis was unable to restrict the periapical diagnosis of AP. Garrido et al. [7] did not specify the type of AP, which may have led to the inclusion of different diagnoses and, consequently, variations in lesion repair.

In the cardiovascular context, the magnitude of change in IL‐1β levels considered clinically relevant is not based on the absolute reduction in this circulating cytokine but on the functional attenuation of the inflammatory pathway that it triggers. IL‐1β acts as an initial mediator of the inflammatory cascade, inducing the production of IL‐6 and subsequently stimulating the hepatic synthesis of hs‐CRP. Thus, the clinical relevance of IL‐1β modulation should be interpreted primarily based on the consistent and sustained reduction in these subsequent inflammatory markers, which reflect the attenuation of systemic inflammation and may be associated with a lower cardiovascular risk [35].

Likewise, there was no association between treatment and reduction in IL‐6 after 6 months (*p* = 0.37) or 1 year (*p* = 0.16). IL‐6 plays an important role in the development of the atherosclerotic process, promoting an increase in vascular inflammation, endothelial dysfunction, and atherosclerotic plaque instability, factors that contribute to the development of cardiovascular diseases [42]. This cytokine is produced during inflammation after the release of TNF‐α and IL‐1β in response to stimuli such as infection and trauma. Furthermore, elevated serum levels of IL‐6 stimulate the production of hs‐CRP [43], with these levels being higher in patients with AP [7, 36]. Therefore, even moderate reductions in IL‐6 may reflect significant attenuation of systemic inflammatory pathways that are relevant to atherosclerosis [43]. Studies have shown that the size of the apical lesion and the presence of symptoms increase the expression of this cytokine [14, 44]. However, among the included studies, two evaluated AP without reporting details of the lesion [7, 12], and one included teeth showing radiographic loss greater than 2 mm at the largest diameter in the AP lesion [26]. These factors may have limited the detection of an association between IL‐6 and endodontic treatment.

Similarly, the results of meta‐analysis revealed no significant association between endodontic treatment and reduction in TNF‐α at 6 months (*p* = 0.91) or 1 year after treatment (*p* = 0.20). This biomarker is involved in the development of atherosclerosis and recruitment of inflammatory cells [45]. Together with IL‐1β and IL‐6, TNF‐α forms a network of cytokines that can affect plaque progression and vascular physiology, a fact that justifies monitoring these biomarkers in the treatment of atherosclerosis [46]. In addition to its proinflammatory activity, TNF‐α also participates in the tissue repair process by contributing to the regulation of bone remodeling and promoting the recruitment and migration of mesenchymal stem cells to the site of injury, where they assist in tissue regeneration [47]. Considering the close biological relationship between TNF‐α and IL‐6 and their synergistic action in the modulation of the inflammatory response and differentiation of osteoclasts and osteoblasts [48], the persistence of systemic levels of these cytokines after treatment may reflect not only residual inflammation but also ongoing physiological repair processes.

In contrast, hs‐CRP, an acute‐phase protein that is considered a reliable marker of cardiovascular risk [49], was significantly reduced after 6 months (*p* < 0.00001) but not after 1 month (*p* = 0.06).

Elevated hs‐CRP levels have been consistently associated with an increased risk of myocardial infarction, stroke, and peripheral artery disease [49]. From a clinical point of view, reductions that alter the risk category (< 1 mg/L: low risk; 1–3 mg/L: moderate risk; > 3 mg/L: high risk) are considered to be associated with a significant change of cardiovascular risk [50]. Thus, reductions in hs‐CRP levels after endodontic treatment may be clinically relevant when they contribute to the change between these risk categories. The lack of significance during short‐term follow‐up (1 month) can be explained by the temporal dynamics of the systemic inflammatory response and the time necessary for complete resolution of the periapical inflammatory process. Since periapical repair is a gradual process that involves cytokine modulation, cell recruitment, and tissue reorganization, it is biologically plausible that the reduction in systemic inflammation becomes more evident only after a few months [33]. This finding suggests that short‐term follow‐up may not be sufficient to detect relevant systemic changes in hs‐CRP.

This review has limitations that are mainly due to the lack of exclusion criteria in the primary studies regarding systemic conditions and potential confounders such as marginal periodontitis and smoking. Several studies did not adequately report the periodontal status of the patients [13, 22, 23, 25, 31, 32] despite the well‐established association between periodontitis and increases in systemic inflammatory biomarkers [39], a fact that compromises the internal validity of the included studies and limits the causal attribution of the observed biomarker changes exclusively to endodontic treatment. Smoking was also an uncontrolled confounding factor in many studies [11, 13, 15, 21, 26–29, 31, 32]. Nicotine is known to induce the expression of biomarkers associated with chronic diseases such as IL‐6, CRP, ICAM‐1, and MCP‐1 [51–53]. Thus, adopting rigorous methodological standardization is essential to enable direct comparisons between studies and reliable extrapolation of their conclusions [54]. Standardized description of the clinical and radiographic assessment of cases, coupled with the systematic use of methodological checklists, is fundamental for reducing bias, increasing transparency, and strengthening the robustness of inferences regarding the effects of endodontic treatment on systemic inflammatory biomarkers. Without such measures, the interpretation of results remains limited, and the formulation of therapeutic recommendations based on solid evidence is compromised [55].

Most studies assessed circulating biomarkers in blood; however, heterogeneity in sample processing may have contributed to the variability of the results. Five studies used plasma [12, 13, 23, 25, 26], and 11 used serum [7, 9, 14, 15, 21, 22, 27, 31]. These differences can affect cytokine measurements because blood contains cells suspended in plasma, while serum is obtained after clotting and centrifugation of the blood sample [56, 57]. During clotting, platelets release a range of inflammatory mediators, which contribute to the increase in cytokine levels during processing [56]. One study measured salivary biomarkers [9] since saliva contains substances that diffuse from the bloodstream [58]. However, the correlation between biomarker levels in saliva and blood varies according to the marker tested and local conditions [59–61]. Therefore, blood remains the gold standard for systemic biomarker assessment [59]. Two studies measured biomarkers in gingival crevicular fluid [24, 29]. However, measurement in gingival crevicular fluid provides only local assessment of biomarkers and is used as a diagnostic tool [24, 62].

The clinical relevance of this systematic review and meta‐analysis should be interpreted with caution since most of the included studies were observational, consisted of small‐scale trials, evaluated different biomarkers, and were classified as high risk of bias and poor quality. Methodological differences and the lack of reported data did not permit meta‐analysis of most biomarkers. Even for the biomarkers included in the meta‐analyses, the number of studies was small, and the small sample sizes contributed to the high heterogeneity in the meta‐analysis. Future studies that use clearer inclusion criteria and longer follow‐ups are necessary to clarify the influence of endodontic treatment on the reduction in these biomarkers.

This systematic review and meta‐analysis synthesized current evidence on the impact of endodontic treatment for AP on levels of inflammatory biomarkers associated with cardiovascular risk. Using GRADE, the overall certainty was classified as low, mainly because of inconsistency across the primary studies, which was characterized by substantial clinical heterogeneity and variability in effect sizes. Some studies reported significant reductions in biomarker levels, while others found no statistically significant differences. In addition, imprecision was observed since most biomarkers were assessed in a small number of studies, which often had small sample sizes and wide CIs.

## 5. Conclusion and Recommendations

Endodontic treatment of teeth with AP was associated with a significant reduction in hs‐CRP at 6 months but not at 1 month. No significant reductions were observed in IL‐6 or TNF‐α at 6 months and 1 year and in IL‐1β at 6 months. These findings should be interpreted with caution since several of the included studies did not adequately control for key confounders such as marginal periodontitis and smoking, which are linked to increased systemic inflammatory burden. Well‐designed clinical trials using rigorous control of these factors, standardized methodologies, and longer follow‐up periods are needed to confirm these results.

## Author Contributions

Carolina Viana Vasco Lyra: conceptualization, data curation, formal analysis, writing–original draft, and writing–review and editing. Jéssica Meirinhos Miranda: data curation, formal analysis, and writing–original draft. Renata de Albuquerque Cavalcanti Almeida: conceptualization, methodology, and formal analysis. Marina da Cunha Isaltino: writing–original draft and visualization. Ana Virginia Silva Vilela: writing–original draft and writing–review and editing. Marleny Elizabeth Márquez de Martínez Gerbi: supervision and writing–review and editing. Diana Santana de Albuquerque: validation, supervision, and writing–review and editing. Natália Gomes de Oliveira: conceptualization, methodology, supervision, and project administration.

## Funding

The authors did not receive support from any organization for the submitted work.

## Ethics Statement

The authors have nothing to report.

## Conflicts of Interest

The authors declare no conflicts of interest.

## Supporting Information

Additional supporting information can be found online in the Supporting Information section.

## Supporting information


**Supporting Information 1** Supporting Table 1. Detailed electronic search strategies were used for each database, including free‐text keywords, MeSH terms, and Emtree terms, combined by Boolean operators “OR” or “AND.” Searches were conducted in April 2025 in the following databases: PubMed, Embase, Web of Science, Scopus, Cochrane Library, and LILACS. No date restrictions were applied.

**Supporting Information 2** Supporting Table 2. Summary of findings and certainty of evidence according to the GRADE approach. This table shows the overall assessment of the certainty of evidence regarding the reduction in inflammatory biomarkers after endodontic treatment in patients with apical periodontitis. The certainty of evidence was rated as low, mainly due to concerns related to risk of bias, inconsistency, and imprecision. No concerns were identified regarding indirectness since the outcome directly corresponds to the reduction in inflammatory biomarkers.

## Data Availability

The data that support the findings of this study are available in the supporting information of this article.
