# Supplementary material for: Impact of Endodontic Treatment of Teeth With Apical Periodontitis on Levels of Inflammatory Biomarkers Associated With Cardiovascular Risk: A Systematic Review and Meta‐Analysis
Source: ScientificWorldJournal. 2026 Apr 23;2026:5896031. doi: 10.1155/tswj/5896031 (PMC13106982; doi:10.1155/tswj/5896031)
Supplement: Supplementary file 1 — Supporting Information 1 Supporting Table 1. Detailed electronic search strategies were used for each database, including free‐text keywords, MeSH terms, and Emtree terms, combined by Boolean operators “OR” or “AND.” Searches were conducted in April 2025 in the following databases: PubMed, Embase, Web of Science, Scopus, Cochrane Library, and LILACS. No date restrictions were applied. [file TSWJ-2026-5896031-s001.zip › Data Bases_2.docx]

| Data Bases | Search Strategy |
| --- | --- |
| Medline | ((((((((("Periapical Disease") ) OR ("Periapical Abscesses")) OR ("Suppurative Periapical Periodontitis")) OR ("Apical Periodontitis")) OR ("Periapical Granulomas")) OR ("Dental Granulomas")) OR (((((("Periapical Diseases"[Mesh]) ) OR ("Periapical Abscess"[Mesh])) OR ("Periapical Periodontitis"[Mesh])) OR ("Periapical Granuloma"[Mesh]))) AND (((((((((("Endodontic Treatment") OR ("Root Canal Treatment")) OR ("Root Canal Therapies")) OR (Apicoectomies)) OR ("Root Canal Procedure")) OR ("Root end Resection")) OR ("Root end Surgery")) OR ("Root Resection")) OR ("Endodontic Retreatment")) OR ((("Root Canal Therapy"[Mesh]) OR ("Apicoectomy"[Mesh])) OR ("Endodontics"[Mesh])))) AND ((((((((((((((((((("Cytokines"[Mesh])) OR ("Biomarkers"[Mesh])) OR ("C-Reactive Protein"[Mesh])) OR ("Inflammation Mediators"[Mesh])) OR ("Interleukins"[Mesh])) OR ("Interleukin-1beta"[Mesh])) OR ("Interleukin-6"[Mesh])) OR ("Interleukin-8"[Mesh])) OR ("Tumor Necrosis Factor-alpha"[Mesh])) OR ("PTX3 protein" [Supplementary Concept])) OR ("Intercellular Adhesion Molecule-1"[Mesh])) OR (ICAM1 protein, human [Supplementary Concept])) OR ("Vascular Cell Adhesion Molecule-1"[Mesh])) OR ("Fibroblast Growth Factor-23"[Mesh])) OR ("Matrix Metalloproteinase 2"[Mesh])) OR ("E-Selectin"[Mesh])) OR ("Lipopolysaccharide Receptors"[Mesh])) OR (((((((((((((((((((((((((((((((((Cytokine) OR ("Surrogate Marker")) OR ("Surrogate Endpoint")) OR ("Immune Marker")) OR ("Immunologic Marker")) OR ("Laboratory Marker")) OR ("Serum Marker")) OR ("Biochemical Marker")) OR ("Mediators of Inflammation")) OR ("C Reactive Protein")) OR ("hs-CRP")) OR ("High Sensitivity C-Reactive Protein")) OR ("High Sensitivity C Reactive Protein")) OR (Interleukin)) OR ("Interleukin 1beta")) OR (Catabolin)) OR ("IL-1 beta")) OR ("Interleukin 1 beta")) OR ("Interleukin 6")) OR ("IL6")) OR ("IL-6")) OR ("Interleukin 8")) OR (IL-8)) OR (IL8)) OR ("TNF-alpha")) OR ("Tumor Necrosis Factor")) OR ("TNFalpha")) OR ("pentraxin 3")) OR ("pentraxin-related protein 3")) OR ("Intercelluar Adhesion Molecule 1")) OR ("ICAM-1")) OR ("VCAM-1")) OR ("MMP 2 Metalloproteinase"))) |
| Embase | ('tooth periapical disease'/exp OR 'dental granuloma' OR 'periapical disease' OR 'periapical diseases' OR 'periapical granuloma' OR 'periapical infection' OR 'periapical lesion' OR 'periapical periodontitis' OR 'tooth granuloma' OR 'tooth periapical disease' OR 'tooth periapical granuloma' OR 'tooth periapical infection' OR 'periapical abscess'/exp OR 'alveolar abscess' OR 'apical abscess (dental)' OR 'dento alveolar abscess' OR 'dentoalveolar abscess' OR 'periapical abscess' OR 'periapical abscesses' OR 'suppurative apical periodontitis' OR 'suppurative periapical periodontitis' OR 'chronic periodontitis'/exp OR 'chronic periodontitis' OR 'periodontitis chronica') AND ('endodontic procedure'/exp OR 'endodontic procedure' OR 'root canal procedure' OR 'root canal therapy' OR 'apicoectomy'/exp OR 'apicoectomy' OR 'root end resection' OR 'root end surgery' OR 'root resection' OR 'endodontics'/exp OR 'endodontics' OR 'endodontic treatment' OR 'root canal treatment'/exp OR 'root canal therapies') AND ('biological marker'/exp OR 'biological marker' OR 'biological markers' OR 'biomarker' OR 'biomarkers' OR 'marker, biological' OR 'disease marker'/exp OR 'disease marker' OR 'surrogate marker' OR 'biochemical marker'/exp OR 'biochemical marker' OR 'marker, biochemical' OR 'immune marker'/exp OR 'c reactive protein'/exp OR 'c reactive protein' OR 'c reaction protein' OR 'c-reactive protein' OR 'creactive protein' OR 'crp' OR 'protein, c reactive' OR 'serum c reactive protein' OR 'high sensitivity c reactive protein'/exp OR 'autacoid'/exp OR 'autacoid' OR 'autacoid agent' OR 'autacoids' OR 'inflammation mediators' OR 'cytokine'/exp OR 'cytokine' OR 'cytokines' OR 'interleukin' OR 'interleukin 1beta'/exp OR 'il 1 beta' OR 'il 1beta' OR 'interleukin 1 beta' OR 'interleukin 1beta' OR 'interleukin-1beta' OR 'catabolin'/exp OR 'interleukin 6'/exp OR 'il 6' OR 'interleukin 6' OR 'interleukin-6' OR 'interleukin 8'/exp OR 'il 8' OR 'interleukin 8' OR 'interleukin-8' OR 'tumor necrosis factor'/exp OR 'tnf alfa' OR 'tnf alpha' OR 'recombinant tumour necrosis factor alpha' OR 'tissue necrosis factor' OR 'tumor necrosis factor' OR 'tumor necrosis factor alfa' OR 'tumor necrosis factor alpha' OR 'tumor necrosis factor-alpha' OR 'tumor necrosis factors' OR 'tumour necrosis factor' OR 'tumour necrosis factor alfa' OR 'tumour necrosis factor alpha' OR 'tumour necrosis factor-alpha' OR 'tumour necrosis factors' OR 'pentraxin 3'/exp OR 'ptx3 protein' OR 'long pentraxin 3' OR 'long pentraxin ptx3' OR 'pentraxin 3' OR 'protein ptx3' OR 'intercellular adhesion molecule 1'/exp OR 'icam 1' OR 'intercellular adhesion molecule 1' OR 'intercellular adhesion molecule-1' OR 'icam1 protein human'/exp OR 'vascular cell adhesion molecule 1'/exp OR 'vcam 1' OR 'vascular cell adhesion molecule 1' OR 'vascular cell adhesion molecule-1' OR 'fibroblast growth factor 23'/exp OR 'fgf 23' OR 'fgf23' OR 'fibroblast growth factor 23' OR 'fibroblast growth factor-23' OR 'gelatinase a'/exp OR 'mmp 2' OR 'mmp2' OR 'gelatinase a' OR 'endothelial leukocyte adhesion molecule 1'/exp OR 'e selectin' OR 'e-selectin' OR 'endothelial leucocyte adhesion molecule 1' OR 'selectin e' OR 'lipopolysaccharide receptor'/exp OR 'lps receptor' OR 'lipopolysaccharide receptor' OR 'lipopolysaccharide receptors') |
| Web of Science | ALL=("Periapical Diseases" OR "Periapical Abscess" OR "Periapical Periodontitis" OR "Periapical Granuloma" OR "Periapical Disease" OR "Periapical Abscesses" OR "Suppurative Periapical Periodontitis" OR "Apical Periodontitis" OR "Periapical Granulomas" OR "Dental Granulomas" ) AND ALL=("Root Canal Therapy" OR Apicoectomy OR Endodontics OR "Endodontic Treatment" OR "Root Canal Treatment" OR "Root Canal Therapies" OR Apicoectomies OR "Root Canal Procedure" OR "Root end Resection" OR "Root end Surgery" OR "Root Resection" OR "Endodontic Retreatment" ) AND ALL=(Cytokines OR Biomarkers OR "C-Reactive Protein" OR "Inflammation Mediators" OR Interleukins OR "Interleukin-1beta" OR Interleukin-6 OR Interleukin-8 OR "Tumor Necrosis Factor-alpha" OR "PTX3 protein" OR "Intercellular Adhesion Molecule-1" OR “ICAM1 protein, human” OR "Vascular Cell Adhesion Molecule-1" OR "Fibroblast Growth Factor-23" OR "Matrix Metalloproteinase 2" OR E-Selectin OR "Lipopolysaccharide Receptors" OR Cytokine OR "Surrogate Marker" OR "Surrogate Endpoint" OR "Immune Marker" OR "Immunologic Marker" OR "Laboratory Marker" OR "Serum Marker" OR "Biochemical Marker" OR "Mediators of Inflammation" OR "C Reactive Protein" OR hs-CRP OR "High Sensitivity C-Reactive Protein" OR "High Sensitivity C Reactive Protein" OR Interleukin OR "Interleukin 1beta" OR Catabolin OR "IL-1 beta" OR "Interleukin 1 beta" OR "Interleukin 6" OR IL6 OR IL-6 OR "Interleukin 8" OR IL-8 OR IL8 OR TNF-alpha OR "Tumor Necrosis Factor" OR TNFalpha OR "pentraxin 3" OR "pentraxin-related protein 3" OR "Intercelluar Adhesion Molecule 1" OR ICAM-1 OR VCAM-1 OR "MMP 2 Metalloproteinase") |
| Scopus | TITLE-ABS-KEY ( "Periapical Disease" OR "Periapical Abscesses" OR "Suppurative Periapical Periodontitis" OR "Apical Periodontitis" OR "Periapical Granulomas" OR "Dental Granulomas" OR "Periapical Diseases" OR "Periapical Abscess" OR "Periapical Periodontitis" OR "Periapical Granuloma" ) AND TITLE-ABS-KEY ( "Endodontic Treatment" OR "Root Canal Treatment" OR "Root Canal Therapies" OR apicoectomies OR "Root Canal Procedure" OR "Root end Resection" OR "Root end Surgery" OR "Root Resection" OR "Endodontic Retreatment" OR "Root Canal Therapy" OR apicoectomy OR endodontics ) AND TITLE-ABS-KEY ( cytokines OR biomarkers OR "C-Reactive Protein" OR "Inflammation Mediators" OR interleukins OR "Interleukin-1beta" OR "Interleukin-6" OR "Interleukin-8" OR "Tumor Necrosis Factor-alpha" OR "PTX3 protein" OR "Intercellular Adhesion Molecule-1" OR "ICAM1 protein, human" OR "Vascular Cell Adhesion Molecule-1" OR "Fibroblast Growth Factor-23" OR "Matrix Metalloproteinase 2" OR "E-Selectin" OR "Lipopolysaccharide Receptors" OR cytokine OR "Surrogate Marker" OR "Surrogate Endpoint" OR "Immune Marker" OR "Immunologic Marker" OR "Laboratory Marker" OR "Serum Marker" OR "Biochemical Marker" OR "Mediators of Inflammation" OR "C Reactive Protein" OR "hs-CRP" OR "High Sensitivity C-Reactive Protein" OR "High Sensitivity C Reactive Protein" OR interleukin OR "Interleukin 1beta" OR catabolin OR "IL-1 beta" OR "Interleukin 1 beta" OR "Interleukin 6" OR il6 OR "IL-6" OR "Interleukin 8" OR "IL-8" OR il8 OR "TNF-alpha" OR "Tumor Necrosis Factor" OR "TNFalpha" OR "pentraxin 3" OR "pentraxin-related protein 3" OR "Intercelluar Adhesion Molecule 1" OR "ICAM-1" OR "VCAM-1" OR "MMP 2 Metalloproteinase" ) |
| Cochrane | ("Periapical Diseases" OR "Periapical Abscess" OR "Periapical Periodontitis" OR "Periapical Granuloma" OR "Periapical Disease" OR "Periapical Abscesses" OR "Suppurative Periapical Periodontitis" OR "Apical Periodontitis" OR "Periapical Granulomas" OR "Dental Granulomas"):ti,ab,kw AND ("Root Canal Therapy" OR "Apicoectomy" OR "Endodontics" OR "Endodontic Treatment" OR "Root Canal Treatment" OR "Root Canal Therapies" OR Apicoectomies OR "Root Canal Procedure" OR "Root end Resection" OR "Root end Surgery" OR "Root Resection" OR "Endodontic Retreatment"):ti,ab,kw AND ("Cytokines" OR "Biomarkers" OR "C-Reactive Protein" OR "Inflammation Mediators" OR "Interleukins" OR "Interleukin-1beta" OR "Interleukin-6" OR "Interleukin-8" OR "Tumor Necrosis Factor-alpha" OR "PTX3 protein" OR "Intercellular Adhesion Molecule-1" OR “ICAM1 protein, human” OR "Vascular Cell Adhesion Molecule-1" OR "Fibroblast Growth Factor-23" OR "Matrix Metalloproteinase 2" OR "E-Selectin" OR "Lipopolysaccharide Receptors" OR Cytokine OR "Surrogate Marker" OR "Surrogate Endpoint" OR "Immune Marker" OR "Immunologic Marker" OR "Laboratory Marker" OR "Serum Marker" OR "Biochemical Marker" OR "Mediators of Inflammation" OR "C Reactive Protein" OR "hs-CRP" OR "High Sensitivity C-Reactive Protein" OR "High Sensitivity C Reactive Protein" OR Interleukin OR "Interleukin 1beta" OR Catabolin OR "IL-1 beta" OR "Interleukin 1 beta" OR "Interleukin 6" OR "IL6" OR "IL-6" OR "Interleukin 8" OR IL-8 OR IL8 OR "TNF-alpha" OR "Tumor Necrosis Factor" OR "TNFalpha" OR "pentraxin 3" OR "pentraxin-related protein 3" OR "Intercelluar Adhesion Molecule 1" OR "ICAM-1" OR "VCAM-1" OR "MMP 2 Metalloproteinase"):ti,ab,kw" (Word variations have been searched) |
| Lilacs | ("Periapical Disease" OR "Periapical Abscesses" OR "Suppurative Periapical Periodontitis" OR "Apical Periodontitis" OR "Periapical Granulomas" OR "Dental Granulomas" OR "Periapical Diseases" OR "Periapical Abscess" OR "Periapical Periodontitis" OR "Periapical Granuloma") AND ("Endodontic Treatment" OR "Root Canal Treatment" OR "Root Canal Therapies" OR Apicoectomies OR "Root Canal Procedure" OR "Root end Resection" OR "Root end Surgery" OR "Root Resection" OR "Endodontic Retreatment" OR "Root Canal Therapy" OR Apicoectomy OR Endodontics) AND (Cytokines OR Biomarkers OR "C-Reactive Protein" OR "Inflammation Mediators" OR Interleukins OR "Interleukin-1beta" OR "Interleukin-6" OR "Interleukin-8" OR "Tumor Necrosis Factor-alpha" OR "PTX3 protein" OR "Intercellular Adhesion Molecule-1" OR “ICAM1 protein, human” OR "Vascular Cell Adhesion Molecule-1" OR "Fibroblast Growth Factor-23" OR "Matrix Metalloproteinase 2" OR "E-Selectin" OR "Lipopolysaccharide Receptors" OR Cytokine OR "Surrogate Marker" OR "Surrogate Endpoint" OR "Immune Marker" OR "Immunologic Marker" OR "Laboratory Marker" OR "Serum Marker" OR "Biochemical Marker" OR "Mediators of Inflammation" OR "C Reactive Protein" OR "hs-CRP" OR "High Sensitivity C-Reactive Protein" OR "High Sensitivity C Reactive Protein" OR Interleukin OR "Interleukin 1beta" OR Catabolin OR "IL-1 beta" OR "Interleukin 1 beta" OR "Interleukin 6" OR IL6 OR "IL-6" OR "Interleukin 8" OR “IL-8” OR IL8 OR "TNF-alpha" OR "Tumor Necrosis Factor" OR "TNFalpha" OR "pentraxin 3" OR "pentraxin-related protein 3" OR "Intercelluar Adhesion Molecule 1" OR "ICAM-1" OR "VCAM-1" OR "MMP 2 Metalloproteinase") |

**Supplementary Table 1.** Detailed electronic search strategies used for each database, including free-text keywords, Mesh terms, and Entree terms, combined by Boolean operators “OR” or “AND”. Searches were conducted in April 2025 in the following databases: PubMed, Embase, Web of Science, Scopus, Cochrane Library, and LILACS. The search was performed in English, with no publication date restrictions.
