# Supplementary material for: Impact of Endodontic Treatment of Teeth With Apical Periodontitis on Levels of Inflammatory Biomarkers Associated With Cardiovascular Risk: A Systematic Review and Meta‐Analysis
Source: ScientificWorldJournal. 2026 Apr 23;2026:5896031. doi: 10.1155/tswj/5896031 (PMC13106982; doi:10.1155/tswj/5896031)
Supplement: Supplementary file 2 — Supporting Information 2 Supporting Table 2. Summary of findings and certainty of evidence according to the GRADE approach. This table shows the overall assessment of the certainty of evidence regarding the reduction in inflammatory biomarkers after endodontic treatment in patients with apical periodontitis. The certainty of evidence was rated as low, mainly due to concerns related to risk of bias, inconsistency, and imprecision. No concerns were identified regarding indirectness since the outcome directly corresponds to the reduction in inflammatory biomarkers. [file TSWJ-2026-5896031-s002.zip › GRADE_1.docx]

| **Certainty assessment** | | | | | | | **№ of patients** | | **Effect** | | **Certainty** | **Importance** |
| --- | --- | --- | --- | --- | --- | --- | --- | --- | --- | --- | --- | --- |
| **№ of studies** | **Study design** | **Risk of bias** | **Inconsistency** | **Indirectness** | **Imprecision** | **Other considerations** | **[Intervention]** | **[Comparison]** | **Relative (95% CI)** | **Absolute (95% CI)** |  |  |
|  | | | | | | | | | | | | |
| Reduction in inflammatory biomarkers (measured using blood samples, saliva, and gingival crevicular fluid | | | | | | | | | | | | |
| 20 | Clinical Trials | Not serious | Serious | Not serious | Serious | None | 642 | None | Not estimable | Not estimable | ⨁⨁◯◯ Low | Important but not critical |

**Supplementary Table 2.** Summary of findings and certainty of the evidence according to the GRADE approach. This table presents the overall assessment of the certainty of evidence regarding the reduction of inflammatory biomarkers after endodontic treatment in patients with apical periodontitis. The certainty of evidence was rated as low, mainly due to concerns related to risk of bias, inconsistency, and imprecision. No concerns were identified regarding indirectness, as the outcome directly corresponds to the reduction in inflammatory biomarkers.
